# A Novel SELEX Based on Immobilizing Libraries Enables Screening of Saxitoxin Aptamers for BLI Aptasensor Applications

**DOI:** 10.3390/toxins14030228

**Published:** 2022-03-21

**Authors:** Rong Zhou, Yun Gao, Chengfang Yang, Xiaojuan Zhang, Bo Hu, Luming Zhao, Han Guo, Mingjuan Sun, Lianghua Wang, Binghua Jiao

**Affiliations:** 1 Department of Biochemistry and Molecular Biology, College of Basic Medical Sciences, Navy Medical University, Shanghai 200433, China; rongzhou1224@126.com (R.Z.); gaoyun2014@sohu.com (Y.G.); nicole20220104@126.com (C.Y.); emilyzhangxj@126.com (X.Z.); zlm19960726@163.com (L.Z.); ggatecnu@163.com (H.G.); sunmj@smmu.edu.cn (M.S.); 2 College of Medicine, Shaoxing University, 900th Chengnan Avenue, Shaoxing 312000, China; 3 Department of Marine Biomedicine and Polar Medicine, Naval Medical Center of PLA, Navy Medical University, Shanghai 200433, China; hb8601@163.com

**Keywords:** saxitoxin, IMC-SELEX, molecular docking, molecular dynamics simulation, aptasensor

## Abstract

Saxitoxin (STX) is one of the potent marine biotoxins that has high rate of lethality. However, there are no effective treatments at present, and the existing detection methods need to be further explored because of ethical problems or technical limitations. In this work, oligonucleotide aptamers toward STX were screened based on immobilizing libraries on Immobilized Metal-Chelate (IMC), such as Ni-NTA Sepharose, and the IMC-SELEX was conducted by the G-quadruplex library and the random library, respectively. Aptamer 45e (from the G-quadruplex library) and aptamer 75a were obtained after optimization, and aptamer 45e turned out to have a higher affinity toward STX. Furthermore, it was found that the hydrogen bonding and the van der Waals forces (VDW) played major roles in the high efficiency and specificity between STX and 45e by means of molecular docking and dynamics simulation. Based on this, aptamer 45e-1 with the *K*_d_ value of 19 nM was obtained by further optimization, which was then used to construct a simple, label-free and real-time optical BLI aptasensor for the detection of STX. This aptasensor showed good reproducibility and stability. In summary, with the advantages of screening aptamers of high efficiency and specificity toward the targets, the proposed IMC-SELEX provides a promising screening strategy for discovering aptamers, which could be used as the potential molecular recognition elements in the fields of biomedicine, food safety and environmental monitoring.

## 1. Introduction

**Figure 1:**
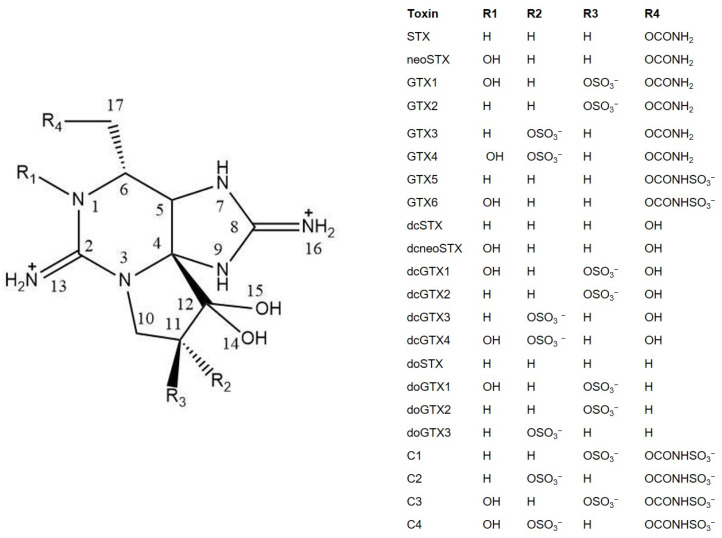
Chemical structures of paralytic shellfish toxins (PSTs).

Paralytic Shellfish toxins (PSTs, Figure 1) are a group of the potent marine biotoxins, known to have a high lethality rate, that are mainly produced by several dinoflagellates and certain cyanobacteria [1,2,3]. However, there is no effective treatment against PSTs and there are some disadvantages associated with certain traditional methods of detection. For example, firstly, the mouse bioassay has poor reproducibility and ethical problems [4]. Secondly, the immunological detection method is cumbersome and prone to cross-reaction [5,6]. Thirdly, the Hydrophilic interaction liquid chromatography-tandem mass spectrometry (HILIC–MS/MS) methods [7,8] have drawbacks such as tedious sample preparation (pre-column method) [9,10] or lack of selectivity for certain congeners (post-column method) [11,12]. However, the appearance of oligonucleotide aptamers has brought new opportunities for the study of PSTs, in particular for saxitoxin (STX).

Aptamers are functional ssDNA or RNA fragments screened from the oligonucleotide libraries through an in vitro evolution process known as Systematic Evolution of Ligands by Exponential Enrichment (SELEX) [13,14,15]. Aptamers have been rapidly applied in various fields ranging from environmental screening, diagnosis, drug delivery to therapy over the past few decades [16,17,18,19,20,21]. In recent years, our group has been constantly concentrating on using aptamers to detect marine biotoxins and achieved promising results [22,23,24,25]. However, there are difficulties in modification, immobilization of small molecules with low molecular weight, and in separating the target-aptamer complexes from the free oligonucleotides. In 2013, Handy et al. [26] obtained the first aptamer APT^STX1^ of saxitoxin through a complicated separation method of immobilized toxin, and Zheng et al. [27] optimized APT^STX1^ through site-directed mutation and truncation to obtain an aptamer M-30f with a special structure of G-quadruplex, which greatly improved the binding affinity of aptamer to saxitoxin. Herein, we speculated that G-quadruplex structures in aptamers might be inclined to form stable a conformation with STX. Sensitive and rapid detection of saxitoxin was realized by combining various detection methods based on aptamer APT^STX1^ or M-30f [28,29,30,31]. However, immobilized targets were especially ill-suited for small molecules, where the immobilization itself is a difficult process due to the small molecular structure and the presence of fewer active sites for conjugation [32]. In other words, the aptamer screened by immobilized targets may be different from the aptamer screened by targets in the natural conformations [33]. Gu [34] and Ha [35] screened two STX aptamers through the immobilized libraries by graphene oxide SELEX (GO-SELEX), overcoming the obstacle that STX was difficult to immobilize. However, in the pre-experiments, we found that graphene oxide (GO) could adsorb the STX-aptamer complex to some extent (Figure S1A,B), which might affect the final screening results. Furthermore, the interaction mechanisms between the aptamers and STX needs to be further clarified.

Immobilized metal–chelate affinity chromatography (IMAC) matrixes are mainly used to adsorb and purify 6×His-tagged fusion protein [36,37]. However, it was reported that IMAC matrixes could strongly adsorb single-stranded nucleic acids through metal ion interactions with aromatic base nitrogen [38,39]. Therefore, IMAC matrixes can be used as candidate solid-phase matrixes to immobilize ssDNA in the screening of aptamers. Among all the IMAC matrixes, Nickle-charged nitrilotriacetic acid (Ni-NTA) matrix is the most commercially available matrix, which makes it the most reliable in quality. Therefore, Ni-NTA matrix can be used as an ideal solid-phase medium for immobilizing libraries when screening STX aptamers. Based on the inducted fit theory [40] and competitive capture mode of affinity, when STX is infinitely close to the ssDNA adsorbed on Ni-NTA matrix, the target molecule induces a significant change in the conformation of corresponding aptamers [41,42,43], weakens the binding force between Ni-NTA matrix and these aptamers, and leads them to release from Ni-NTA columns and then aptamers binds specifically with STX.

In the present study, a novel, simple but efficient screening method named Immobilized Metal–Chelate SELEX (IMC-SELEX) was established in the screening of STX aptamers. The IMC-SELEX strategy used Ni-NTA to immobilize ssDNA without immobilizing small molecule targets. Thus, it avoided the influence of the structural modifications of small molecule targets during immobilization, which allowed toxins to maintain their native structure. Through IMC-SELEX, we obtained aptamers of STX with high affinity. On the basis of obtaining the structural information of interaction between aptamers and the target STX, we further optimized the preliminary optimized aptamer to further improve its affinity. Finally, we developed the final optimized aptamer into a BLI-based aptasensor. This is the first time to use IMC-SELEX as the screening strategy for aptamers, and this strategy can be extended to the screening aptamers of other small molecular targets. In addition, the novel aptasensor established in this work can be used as a reliable, rapid and low-cost tool for the detection of STX, and the high-affinity aptamers for STX obtained by IMC-SELEX can be used as novel molecular recognition elements for the development of other types of aptasensors.

## 2. Results and Discussion

### 2.1. Selection of Aptamers by IMC-SELEX

**Figure 2:**
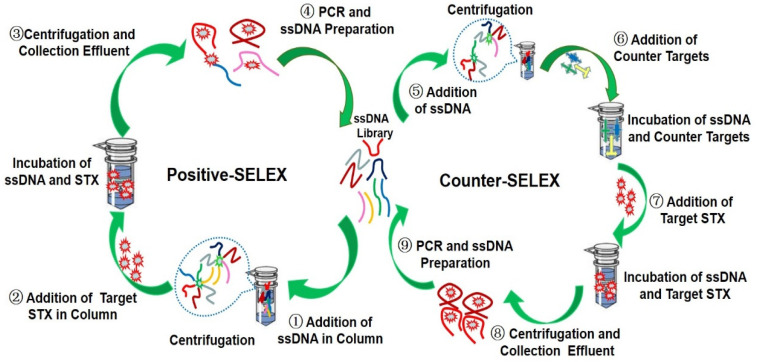
Processes of IMC-SELEX. Positive SELEX (**left**) and counter SELEX (**right**).

To test the ability of the Ni-NTA protein purification centrifugal column (2 mL kit) to adsorb ssDNA, we incubated the column with different concentrations of ssDNA, and tested the remaining ssDNA content of the supernatant. We found that this column had a strong adsorption capacity for ssDNA as previously reported literature [38]. The adsorption of per column (2 mL kit) almost reached 100% for 200 pmol ssDNA and 97% for 1 nmol ssDNA (Figure S1C). Therefore, the Ni-NTA column can be used as a medium to immobilize ssDNA during aptamer screening. Because of the strong adsorption capacity of the medium, the recovery rate was low throughout the screening process. However, the recovery rate showed an upward trend (Figure S1D). Combined with the affinity between STX and the whole library by means of BLI detection, it was found that the *K*_d_ value of the second round and third round increased significantly compared with that of the first round, and the *K*_d_ value of the fifth round and the sixth round tended to be stable (Table S1), so the ssDNA in effluent of the sixth round were cloned and sequenced, and the screening flow chart was shown in Figure 2. A total of 80 sequences were selected from the random library and the G-quadruplex library respectively (Table S1). The similarity and homology of sequences were compared and analyzed by Clustal X 2.1 software and the secondary structure of them were simulated and analyzed by the mfold web server online tool. After that, the sequences with lower free energy when forming a secondary structure were selected as candidate aptamers. At the same time, the online analysis software of G-quadruplex was used to score, and the aptamers with higher scores of G-quadruplex were selected preferentially. Finally, 11 sequences (STX-R-) and 10 sequences (STX-G4-) were selected as candidate aptamers from the random library and the G-quadruplex library, respectively (Table S1).

Since the first application of SELEX about thirty years ago, this method has undergone extensive modifications and improvements [44]. Although the conventional target-immobilized method has many successful screening applications [16], many small molecules are not suitable for this method because of the structural modifications they needed when they are immobilized onto solid-phase medium. These modifications may change their natural structures. In this way, the screened aptamers will not be able to recognize the natural structure of the target [22,45]. As a relatively new method, Capture-SELEX does not need to immobilize the target onto solid-phase medium, thus the problem of structural changes caused by target modification is solved [45,46]. However, it is necessary to design a suitable docking sequence when applying this method and the docking sequence must bind stably and specifically to the capture oligos [45,46]. On the one hand, this method requires a lot of attempts when applied to certain targets. On the other hand, the docking sequence may interfere with the binding of aptamer sequence to targets in Capture-SELEX. In this work, chelated soft metal ions interact with nitrogen on aromatic ring of base of nucleic acids, and this interaction can serve as the basis of IMC-SELEX method [38,39]. This interaction mode is different from that of Capture-SELEX. The relatively low recovery rates throughout the screening process indicate that when applying the IMC-SELEX method, the ssDNA library can be very firmly bound to the solid phase medium, which can reduce non-specific elution during screening, thereby greatly improving the efficiency of screening. However, the problem of non-specific adsorption of the targets to the IMC-SELEX medium still needs to be considered. For example, recombinant proteins containing histidine tags or some natural proteins with continuous histidine can be adsorbed by the IMC medium and may be not suitable for the IMC-SELEX. Therefore, as an alternative to Capture-SELEX, the IMC-SELEX method may have certain advantages when screening specific targets.

### 2.2. Identification of Affinity and Specificity of STX and Aptamer

The BLI technique was used to determine the binding affinity of the 21 candidate aptamers (including 11 sequences selected from the random library and 10 sequences selected from the G-quadruplex library) to STX (5 μM). It was found that STX-R-75 (*K*_d_: 209.4 nM, Table S1) in the random library and STX-G4-45 (*K*_d_: 42.6 nM, Table S1) in the G-quadruplex library had the highest *K*_d_ value, respectively. Additionally, neither of these two aptamers combined with STX analogues (GTX1/4, neoSTX), which indicated that these two aptamers bound STX with high affinity and specificity, and the affinity of STX-G4-45 from the G-quadruplex library was higher than that of STX-R-75 from the random library. However, none of the aptamers with G-quadruplex structure were screened in the random library. We suspected that the random library could also screen out aptamers with G-quadruplex structures if we increased the number of screening rounds and screening pressure. At present, series of aptamers of G-quadruplex sequences have been derived for different targets because of additional advantageous attributes of G-quadruplex with thermodynamic and chemical stability, high specificity of interaction with the target, and the unique folding properties leading to diversity conformations of library [47,48,49]. Therefore, we speculate that when the library contains a large proportion of G-rich sequences, the possibility of obtaining high-affinity aptamers will increase. Thus, under the same conditions, compared with the completely random library, aptamers with higher affinity are easier to obtain from the G-quadruplex library.

### 2.3. Truncation and Optimization of Aptamers

**Figure 3:**
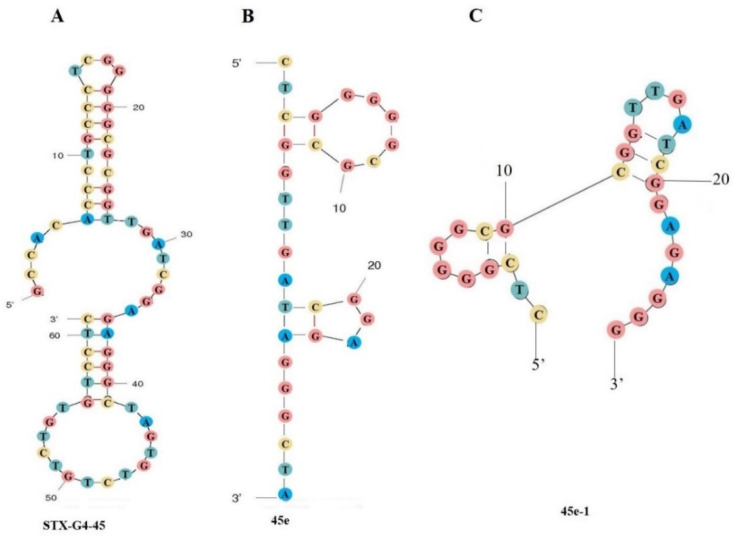
Secondary structure of aptamers. (**A**) STX-G4-45; (**B**) 45e; (**C**) 45e-1.

**Figure 4:**
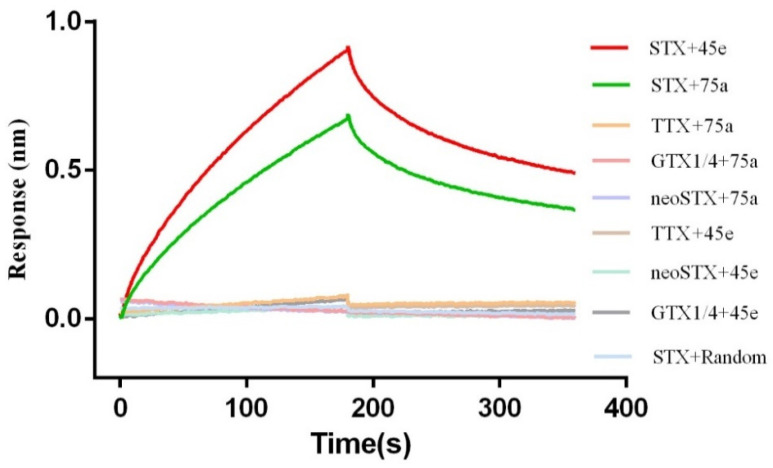
Characterization of affinity and specificity of aptamer 75a and aptamer 45e for STX. The red and green line represent the interaction curves of aptamers 45e and 75a with STX, respectively. The blue line represents the interaction curve of a random sequence with STX. The yellow, pink, purple, brown, light green and grey lines represent the interaction curves of aptamer 75a with TTX, 75a with GTX1/4, 75a with neoSTX, 45e with TTX, 45e with neoSTX, 45e with GTX1/4, respectively. All toxins are diluted to 5 µM with buffer.

The secondary structures of the two aptamers were predicted and analyzed by the mfold web software; some special stem–loop and hairpin structures may be the main sites of interaction between the aptamers and the target molecules [50]. Thus, aptamer STX-G4-45 (Figure 3A) was truncated and optimized to form 45a, 45b, 45c, 45d (Figure S2) and 45e (Figure 3B). The aptamer STX-R-75 was truncated and optimized to form 75a, 75b, and 75c (Figure S3). It was found that the GTG of STX-R-75 downstream primer and the CTC of STX-G4-45 upstream primer might participate in the binding with STX, or have the function of stabilizing the binding of the aptamers to STX. For aptamer STX-G4-45, we also mutated the partial bases (G20, G21, G25, G26, G33, G34, G38 and G39) that might form the G-quadruplexes. We found that the affinity of the aptamer to STX decreased obviously or the aptamer failed to bind to STX (Table S1), so it was speculated that these bases might directly participate in the combination with STX or stabilize the combination of aptamer and STX. Finally, we optimized the aptamers by means of truncation, preliminarily explored the interaction sites between the aptamers and STX. In this process we obtained the aptamer 45e with a *K*_d_ value of 21.2 nM which was improved by 2-fold versus that of STX-G4-45, and aptamer 75a with a *K*_d_ value of 136 nM which was improved by 1.5-fold versus that of STX-R-75. With other tested toxins, no affinity was observed in combination with aptamer 45e and aptamer 75a, and the affinity of 45e (red) and STX was higher than that of 75a (green) and STX in buffer (Figure 4), moreover, 45e also has excellent specificity for binding to STX in seawater samples (Figure S4). Therefore, the aptamer 45e was chosen for subsequent research.

### 2.4. Molecular Docking and Molecular Dynamics Simulations (MDS)

#### 2.4.1. 3-D Structure Modeling of Aptamer 45e

**Figure 5:**
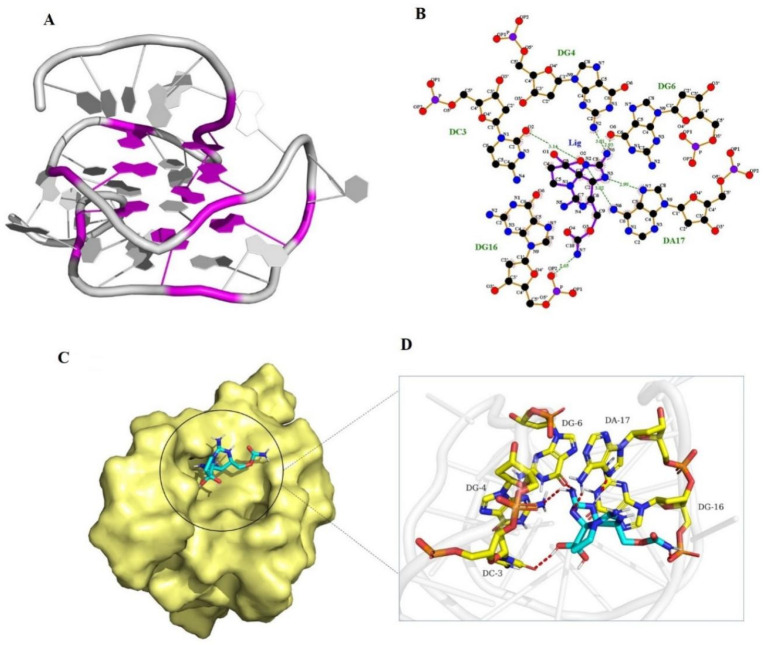
Complex of aptamer 45e with STX. (**A**) 3-D structure model of 45e. (**B**) The 2-D binding mode of 45e with STX. (**C**) The binding model of STX on molecular surface of 45e. STX is cyan (crimson represents the O atom), and the molecular surface of 45e is pale yellow in color. (**D**) The 3-D binding mode of 45e with STX. STX is cyan (crimson represents the O atom), the surrounding residues in the binding pockets are colored in yellow (light red represents the P atom), and the backbone of the receptor is depicted as white cartoon with transparency.

The 3-D structure model of aptamer 45e is shown in Figure 5A. Quadruplex-forming G-Rich Sequences (QGRS) were highlighted with purple color and aptamer 45e forms QGRS area: G6, G7, G12, G13, G20, G21, G25, G26.

#### 2.4.2. Docking and Molecular Dynamics Simulation of Aptamer 45e with Saxitoxin

**Figure 6:**
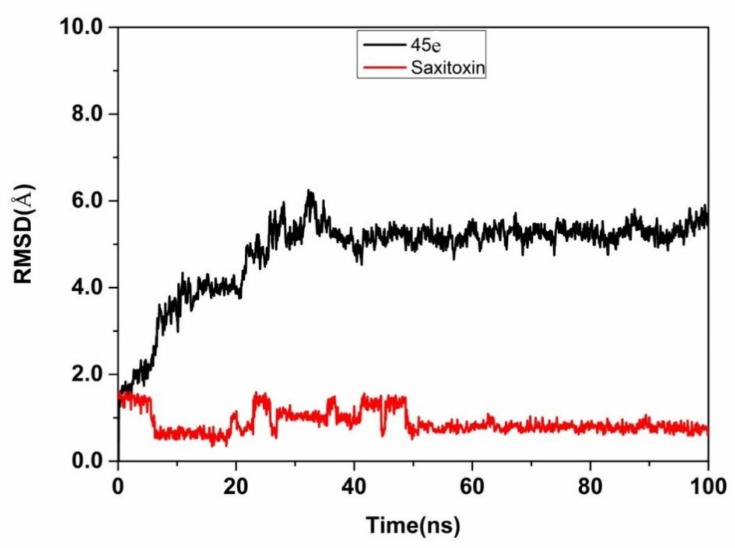
RMSD of aptamer 45e or saxitoxin during the 100 ns simulation.

The docking score was −8.3 kcal/mol. The selected docked pose of complexes was optimized by means of all-atom, explicit water MD simulations. As shown in Figure 6, the root means square deviation (RMSD) of the backbone of aptamer 45e was less than 6.0 angstrom, that of STX was less than 2.0 angstrom, and the system achieved equilibrium within the simulation time, which suggested that the force field and simulation protocols were adequate. The final stable complex of 45e with STX and their respective interactions are shown in Figure 5B–D and Figure S5. Hydrogen bond interactions were formed between 45e with STX. The sixth nitrogen atom of 45e, regarded as a hydrogen bond donor, formed a hydrogen bond with the second nitrogen atom of DG4 and the sixth oxygen atom of DG6, respectively. The seventh nitrogen atom of 45e, regarded as a hydrogen bond donor, formed a hydrogen bond with the OP2 oxygen atom of DG16. The third nitrogen atom of 45e, regarded as a hydrogen bond donor, formed a hydrogen bond with the seventh nitrogen atom of DA17. The second oxygen atom of 45e, regarded as a hydrogen bond donor, formed a hydrogen bond with the second oxygen atom of DC3. The second nitrogen atom of 45e, regarded as hydrogen bond acceptor, formed a hydrogen bond with the sixth nitrogen atom of DA17. Furthermore, VDW interactions were formed among STX and the surround bases. These interactions mainly contributed to the binding energy between 45e with STX and this result was consistent with the decreased affinity of aptamer to STX after partial base truncation. The C base in the upstream primer was indeed directly involved in the binding of the aptamer to STX. Although those bases of G7, G12, G13, G20, G21, G25 and G26, which could form G-quadruplexes, did not directly interact with STX, the affinity of the aptamer to STX decreased or the aptamer failed to bind to STX after mutating these G bases (Table S1). We suspected that these G bases were beneficial to the formation of more stable conformation for the binding of aptamers and STX.

The binding energy (∆G_total_) of 45e with STX was calculated using the MM-GBSA method which is shown in Table 1. The contribution to the binding free energy (∆G_total_) from the VDW and electrostatic interactions was represented by ∆E_vdw_ and ∆E_ele_. The polar and nonpolar solvation energy contributions to ∆G were represented by ∆G_polar_ and ∆G_nonpolar_, respectively. The binding of both 45e-STX was largely governed by electrostatic interactions because ∆E_ele_ has the largest negtive value. ∆G_polar_ was not conducive to binding, as the value was positive, while ∆G_nonpolar_ was favorable, which led to an overall favorable binding energy. The binding free energy upon 45e-STX was computed to be −23.38 ± 0.53 kcal/mol in aqueous environments.

#### 2.4.3. Further Optimization of Aptamer 45e

Through molecular dynamics simulation, the bases were further truncated at the first and last ends of aptamer 45e (*K*_on_ of 4.95 × 10^5^ 1/Ms, *K*_dis_ of 1.05 × 10^2^ 1/s, *K*_d_ of 21.2 nM), whereby the aptamer 45e-1 was obtained (Figure 3C) with *K*_on_ of 9.92 × 10^5^ 1/Ms, *K*_dis_ of 1.89 × 10^2^ 1/s, *K*_d_ of 19 nM. The *K*_on_ of aptamer 45e-1 was improved by 2-fold versus that of 45e and the *K*_dis_ of aptamer 45e-1 was improved by 1.8-fold versus that of 45e. In addition, we found that the binding rate was improved when the C and T bases at the front end of 45e-1 and the last G base of 45e-1 were removed; however, the dissociation rate was also accelerated. Practically, we preferred the mode of fast binding and slow dissociation on BLI analysis; therefore, 45e-1 was used to construct the aptamer biosensor.

### 2.5. Microscale Thermophoresis (MST)

**Figure 7:**
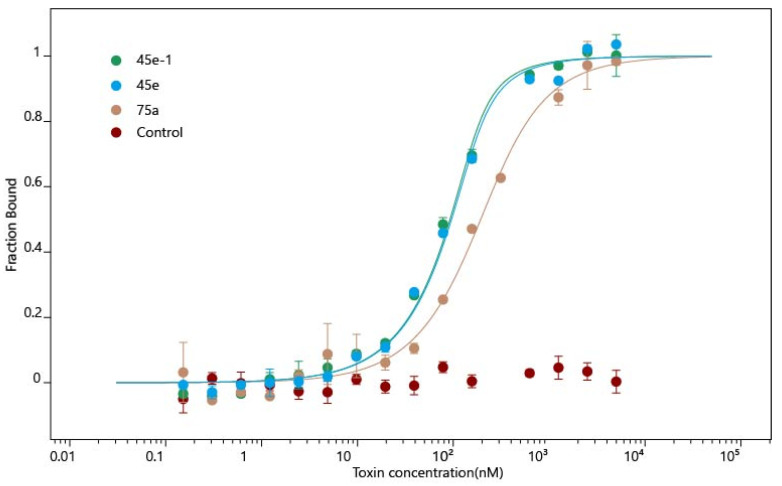
MST experiment for aptamers. MST experiments for 45e-1 (green), 45e (blue), 75a (yellow), and Control (random sequence, red). The *K*_d_ values of 45e-1, 45e and 75a are 17.33 ± 4.84 nM, 20.78 ± 5.70 nM and 117.91 ± 21.51 nM, respectively. The control aptamer shows no binding affinity for STX.

The results of binding affinity between STX and aptamers by MST were consistent with the previous BLI results. Corresponding *K*_d_ values of 45e-1, 45e and 75a are 17.33 ± 4.84 nM, 20.78 ± 5.70 nM and 117.91 ± 21.51 nM, respectively, and the control aptamer shows no binding affinity for STX (Figure 7). Meanwhile, those results also further validated the previous results of molecular docking and molecular dynamics simulations.

### 2.6. BLI Aptasensor for STX Detection

**Figure 8:**
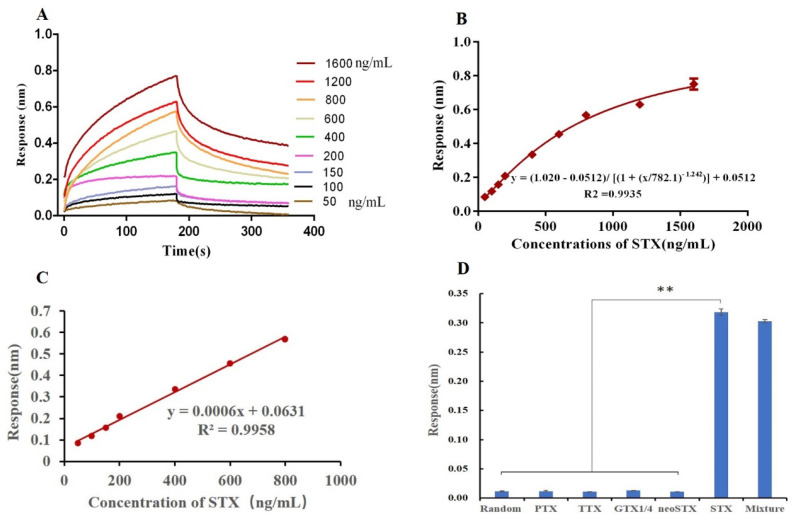
The performance parameters of aptasensors for STX. (**A**) Response values for this aptasensor after the addition of STX at different concentrations (50 ng/mL–1600 ng/mL). (**B**) The calibration curve for STX (50 ng/mL–1600 ng/mL). (**C**) The linear range of the calibration curve for STX (50 ng/mL–800 ng/mL). (**D**) Specificity of the aptasensor with PTX, TTX, GTX1/4, neoSTX and STX (each at 1 µM), and the mixture (STX at 1 µM, and the other toxins at 5 µM each), respectively. ** *p* < 0.01.

Based on nucleic acid aptamer 45e-1, we constructed an aptamer biosensor (aptasensor) for STX (Figure S6). As shown in Figure 8A, with increasing STX concentration, the optical thickness and mass density of biolayer surface gradually increased, and the wavelength changed more obviously, eventually leading to an increase in response value [51]. The experiment was repeated three times for each concentration, and an S-shaped calibration curve was drawn with the concentration as the abscissa and the response value as the ordinate. The logistic five-parameter equation corresponding to the calibration curve is as follows: y = (Rmax − Rmin)/[(1 + (x/EC50) ^b^)] + Rmin, where Rmax and Rmin are the maximum and minimum response values, EC50 is the STX concentration corresponding to half of the maximum response value and b represents the slope of the curve. Following substitution of the experimental data, the following equation was obtained: y = (1.020 − 0.0512)/[(1 + (x/782.1)^−1.242^)] + 0.0512, and the correlation coefficient R^2^ was 0.9935 (Figure 8B). In the concentration range of 50−800 ng/mL, this aptasensor was able to achieve good linear detection. The linear regression equation is y = 0.0006x + 0.0631 (Figure 8C), and the correlation coefficient is 0.9958. According to the detection-limit calculation method in IUPAC, the LOD of this aptasensor was 0.5 ng/mL (S/N = 3; blank sample n = 20). Additionally, this aptasensor was used to measure STX at 100 ng/mL a total of seven times, and the Coefficient of Variation (CV) value at this concentration was calculated to be 3.05%. These results show that this aptasensor has high repeatability.

This aptasensor was used to detect PTX, TTX, GTX1/4, neoSTX and STX (each at 1 µM). The aptasensor showed no affinity to the first four toxins, and the response value produced by these toxins was correspondingly much lower than that produced by STX (Figure 8D). The corresponding response values of PTX, TTX, GTX1/4, neoSTX and STX are 0.0118 nm, 0.0107 nm, 0.0129 nm, 0.0106 nm and 0.3179 nm, respectively. Additionally, we also detected the mixed samples containing five toxins (STX at 1 µM and the other toxins at 5 µM each), and the response value (0.3028 nm) was not obviously different from (*p* > 0.05) that produced by the STX samples alone (Figure 8D). These results showed that this aptasensor was of high specificity and would not be interfered by other toxins such as PTX, TTX, GTX1/4 and neoSTX.

Additionally, we detected and analyzed STX with different concentrations in seawater (3 independent samples), and the results showed that seawater has no obvious interference in the detection results, the recovery rate was 97–106%, and the Coefficient of Variation (CV) value (CV cut-off value in most commonly is 15% [52]) was relatively low (2.4–6.7%), the data are shown in Table 2, and indicate that this aptasensor with excellent stability has the potential to detect STX in complex systems.

Currently, researchers are exploring more and more methods for detecting STX. We summarize the different STX detection methods in Table 3. The HILIC-MS/MS [7,8], Precolumn oxidation HPLC [9], and Postcolumn oxidation HPLC [11,12] methods are expensive and complex to perform. The ELISA detection [6] method is cumbersome (>1 h) and prone to cross-reaction. Although some methods have lower detection limits, such as Electrochemical Ti3C2Tx [53]: 0.03 nM (0.01ng/mL), Colorimetric aptasensor [54,55]: 42.46–142.3 pM (0.01–0.04 ng/mL), Fluorescence assay [34]: 0.39 ng/mL, Electrochemical aptasensor [28,56]: 0.11–0.28 ng/mL, SE aptasensor [31]: 0.11 ng/mL, the detection limit of the sensor (0.5 ng/mL) obtained from this work is significantly lower than the maximum permitted limit of STX (3 ng/mL, toxicity equivalents) in drinking water, which was standardized by Australia, Brazil, and New Zealand [29,53]. Furthermore, compared with the other detection methods, this aptasensor has unique merits. Firstly, the new aptasensor established in this work inherits the consistent advantages of biosensors, which are label free, operate in real time, and are inexpensive [29]. Secondly, this method is relatively simple and can complete the detection and analysis of STX in 10 min without professional operation skills. Thirdly, the sensor is of high specificity and repeatability. Most importantly, the aptamer has higher affinity. We obtained the aptamer 45e-1 with a *K*_d_ value of 19 nM, which was an improvement of about 200-fold versus that of APT^STX1^ [26] (3840 nM). Compared with the aptamer (50.75 ± 14.97 nM) obtained by GO-SELEX, the affinity was also improved by about 2 times. However, none of the technologies are perfect. For this BLI aptasensor, the deficiency is that the detection of complex samples from harsh environments may require pretreatment. In the future, we will further optimize the detection performance of the sensor in terms of aptamer affinity and stability, sensor detection process, etc. Therefore, the novel aptasensor established by this work may be used as an alternative candidate tool for STX detection in the fields of food safety, environmental monitoring and biomedicine.

## 3. Conclusions

In conclusion, a novel screening method named IMC-SELEX was established in this work. The strong adsorption capacity of Ni-NTA matrix to ssDNA made the recovery rate very low throughout the screening process, which might ensure the effective enrichment of specific aptamers. The high affinity of aptamer G4-45 (*K*_d_: 42.6 nM) and R-75 (*K*_d_: 209.4 nM) obtained by IMC-SELEX confirmed the effectiveness of this strategy. Therefore, the IMC-SELEX expanded a new approach especially for the screening of aptamers for small molecules.

Furthermore, the aptamer 45e with improved affinity (*K*_d_: 21.2 nM) was obtained by truncation of G4-45. Based on the structural information of 45e obtained by molecular docking and molecular dynamics simulations, the aptamer was further optimized to generate 45e-1(*K*_d_: 19 nM). 45e-1 was used to develop a BLI-based aptasensor with the LOD of 0.5 ng/mL. This aptasensor has the characteristics of high sensitivity, good repeatability, simple operation, and low cost. This aptasensor may be used as an alternative tool for STX detection in preventing food poisoning or effectively monitor the seawater environment in the future. In addition, the high-affinity aptamer 45e-1 obtained in this work may be further developed into other types of aptasensors in the future.

## 4. Material and Methods

### 4.1. Materials and Reagents

All ssDNA libraries were synthesized by Sangon Biotech (Shanghai, China). Saxitoxin (STX), Neosaxitoxin (neoSTX), Tetrodotoxin (TTX), Gonyautoxin 1/4 (GTX1/4) and palytoxin (PTX) were purchased from Taiwan Algal Science Inc. Binding buffer (20 mM Tris- HCl, 100 mM NaCl, 2 mM MgCl_2_, 5 mM KCl, pH 7.5) was obtained from Tiandz (Beijing, China). The Ni-NTA Spin Kit (2 mL) and A QIAEX^®^ II Gel extraction kits were purchased from Qiagen (Frankfurt, Germany). The Qubit^®^ ssDNA assay kits were procured from Thermo Fisher Scientific (Chelmsford, MA, USA). GoTaqHot^®^ Start colorless master mix was obtained from Promega Corporation (Fitchburg, WI, USA). The 20 bp DNA Ladder (Dye Plus) was purchased from TaKaRa Bio, Inc. (Dalian, China) and SSA sensor chips were purchased from ForteBio (Shanghai, China).

### 4.2. Selection of Aptamers In Vitro

#### 4.2.1. Design of Screening Libraries

Zheng et al. [27] obtained an STX aptamer with G-quadruplex structure having higher affinity after mutation, and there have been many related studies on the aptamer of the G quadruplex based on uniquely folding properties and relatively stable spatial conformation [47,48,49]. Therefore, we assumed that G-quadruplex structures may be conducive to the combination of STX and aptamers. Accordingly, a G-quadruplex library (GCCACACCCTGCCCTCNNNGGNNNNGNNNNNNGNNNNGGGNNNG TGTCTGTCTGTGTCCTC) with immobilized partial of G bases and a random library (GCCACACCCTGCCCTC-N24-GTGTCTGTCTGTGTCCTC) were designed as initial screening libraries, respectively.

#### 4.2.2. Selection of Aptamers by Ni-NTA Column

(1) Ni-NTA columns were balanced with 600 μL screening buffer (20 mM tris-HCl, 100 mM NaCl, 2 mM MgCl_2_, 5 mM KCl, pH 7.5). (2) In the first round, 1 nmol of ssDNA was dissolved in 600 μL of screening buffer. Following heating at 95 °C for 10 min, cooling quickly down 4 °C for 5 min and room temperature for 5 min, the processed ssDNA was added to a well-balanced Ni-NTA column and centrifuged at 270 g for 10 min. The residual ssDNA in the effluent was quantified by Qubit^®^ 2.0 Fluorometer. The Ni-NTA column was washed with 600 μL screening buffer three times. (3) A total of 200 pmol STX was added to the Ni-NTA column, and the column was shaken up and down by means of a shaker, after which the column was placed on a shaker for incubation at room temperature for one hour. Following incubation, the column was centrifuged at 270 g for 10 min and the effluent was taken to quantify. To reduce the possible loss of initial libraries, steps (1)–(3) were repeated twice in the first round, and 1 nmol of initial library was added each time. Finally, the effluent obtained from three times of centrifugation was mixed. (4) The ssDNA in mixed effluent was amplified by PCR, purified by 12% polyacrylamide gel electrophoresis and recovered by Qiagen gel recovery kits. The PCR parameters were set as follows: 95 °C for 5 min, followed by 18–22 cycles of 95 °C for 30 s, 62 °C for 45 s, 72 °C for 30 s, and a final elongation step of 5 min at 72 °C. In this screening, the first round of PCR was 22 cycles, the second round was 20 cycles, and there were 18 cycles thereafter. The screening library of next round was then started with 200 pmol ssDNA. (5) The recovered ssDNA libraries in the first three rounds were amplified by PCR using upstream primers with biotin, after which the ssDNA libraries with biotin were recovered and purified. BLI was applied to measure the affinity between the libraries with biotin and STX. (6) In the fourth round, reverse screening was applied by means of several toxins (TTX, GTX 1/4, neoSTX) having properties similar to STX. Then, after ssDNA was adsorbed by the Ni-NTA column, toxin mixtures (TTX, GTX 1/4, neoSTX, 60 pmol respectively) were added, incubated at room temperature for one hour, and centrifuged at 270 g for 10 min. The Ni-NTA column was washed twice with 600 μL screening buffer. Subsequently, 200 pmol STX was added and steps (3) and (4) were repeated. (7) After six rounds of screening, the recovery rate did not increase significantly, and the *K*_d_ values of libraries measured by BLI had not increased significantly at the same time; then, the screening was stopped.

#### 4.2.3. Clonal Sequencing to Select the Target Aptamer

The ssDNA in effluent from the sixth round was amplified into dsDNA with normal upstream primers and downstream primers without additional modification, and the samples were sent to Sangon Biotech (Shanghai, China) for cloning and sequencing. The sequencing results were analyzed by Clustal X 2.1 software, the secondary structure of the aptamers was the simulated by the mfold web server (http://mfold.rit.albany.edu/?q=mffold or https://sg.idtdna.com/UNAFold?, accessed on 4 January 2021), and the sequences obtained from the G-quadruplex library were analyzed and compared by the online analysis software of G-quadruplex (https://bioinformatics.ramapo.edu/QGRS/index.php, accessed on 4 January 2021). Eleven and ten sequences were selected from the random library and the G-quadruplex library, respectively. The candidate sequences were applied to identify the binding affinity between the aptamers and STX in the next step.

### 4.3. Identification of Affinity and Specificity of STX and Aptamer

Biolayer interferometry (BLI) is a real-time optical analytical technique for reflecting interactions between the solidified ligands and the target molecules [58,59]. Fiberoptic biosensors can monitor changes in the optical thickness of the sensor layer that occur with biological interaction [51]. Thus, BLI can identify the affinity between candidate aptamers and STX or its analogues (TTX, GTX1/4, neoSTX) without labeling. The aptamers with biotin at the 5’end were pretreated (95 °C 10 min, ice bath 5 min, room temperature 5 min) before BLI experiments and connected to the chip probes coated with super streptavidin (SSA), and then reacted with STX solution. Aptamer solution, STX solution and buffer (20 mM Tris- HCl, 100 mM NaCl, 2 mM MgCl_2_, 5 mM KCl, pH 7.5) were added to different columns of 96-well plate and the OctetRED 96 system program was set as follows: (1) sensor balance (1 min); (2) aptamer coupling (2 min); (3) sensor rebalancing (1 min); (4) STX association (3 min); (5) STX dissociation (3 min). With Octet data analysis software CFR Part 11 Version 9.x, the binding-response data were fitted and analyzed by a 1:1 binding mode, so as to obtain the association–dissociation curve of aptamers and the target molecule STX as well as various kinetic parameters, including association rate constant *K*_on_, dissociation rate constant *K*_dis_ and affinity constant *K*_d_.

### 4.4. Molecular Docking and Molecular Dynamics Simulations (MDS)

#### 4.4.1. 3-D Structure Modeling of G-Quadruplex 45e

The sequence was submitted to the QGRS Mapper [60], a web service for predicting G-quadruplexes in nucleotide sequences. The predicted sequences of G-quadruplexes were then submitted to Protein Data Bank (PDB) to find similar sequences of G-quadruplexes with 3-D structures as templates. The template structures (PDB code: 6KVB) were identified to build 3-D models of aptamer 45e. The generated 3-D models were further minimized using the Tinker molecular modeling package [61], with a steepest-descent search on the Amber’99 force-field [62].

#### 4.4.2. Docking of Aptamer 45e with Saxitoxin

Docking studies were conducted using the Autodock Vina program [63] and performed to obtain a population of possible conformations and orientations for the STX at the binding sites. The aptamers were converted to the PDBQT file that contained a structure with hydrogens in all polar residues. All bonds of STX were set as rotatable. All calculations for aptamer-immobilized and STX-flexible docking were done by means of the Lamarckian Genetic algorithm (LGA) method. A grid box with sufficient dimensions to cover the entire DNA structure was prepared. Then, upon completion of the docking search, the best conformation with the lowest binding energy was chosen. The complexes of aptamer 45e with STX after docking were optimized by means of MD simulation.

#### 4.4.3. Molecular Dynamics (MD) Simulation

The complexes of aptamer 45e with STX after docking were optimized by means of MD simulation. The hydrogen atoms of STX were optimized by means of Gaussian09 package at the level of HF/6-31g*. The partial atomic charges were then calculated by the restrained electrostatic potential (RESP) [62] charge from the calculation with Gaussian09 package at the HF/6-31g* level.

The complexes were neutralized with the addition of sodium/chlorine counter ions and solvated in a cuboid box of TIP3P water molecules with solvent layers 10 Å between the box edges and solute surface. All MD simulations were performed using AMBER16 [64]^.^ The AMBER OL15 force fields were applied and the SHAKE algorithm was used to restrict all covalent bonds involving hydrogen atoms with a time step of 2 fs. The particle mesh Ewald (PME) method was employed to treat long-range electrostatic interactions. For the solvated system, two steps of minimization were performed prior to the heating step. The first 4000 cycles of minimization were performed with all heavy atoms restrained with 50 kcal/(mol·Å2), whereas the solvent molecules and hydrogen atoms were free to move. Then, non-restrained minimization was carried out involving 2000 cycles of steepest descent minimization and 2000 cycles of conjugated gradient minimization. Subsequently, the whole system was first heated from 0 K to 300 K in 50 ps using Langevin dynamics at a constant volume, and was then equilibrated for 400 ps at a constant pressure of 1 atm. A weak constraint of 10 kcal/ (mol·Å2) was used to restrain all the heavy atoms during the heating steps. Periodic boundary dynamics simulations were conducted for the whole system with an NPT (constant composition, pressure, and temperature) ensemble at a constant pressure of 1 atm and 300 K in the production step. In production phase, 100 ns simulation was performed. The trajectories were further analyzed by means of Cpptraj. The binding free energy of the complexes was calculated using the MM-GBSA method.

### 4.5. Microscale Thermophoresis (MST)

Furthermore, the binding affinity between STX and aptamers were measured by mean of microscale thermophoresis (MST) [65] using 30% excitation power and 40% IR-laser power (Monolith NT.115 system, NanoTemper Technologies, München, Germany). STX solution was twofold diluted and equal volumes of 6-FAM labeled aptamers at the 3′end (300 nM) was added. The final concentrations of STX ranged from 0.15 to 5000 nM, with a constant aptamer concentration of 150 nM in the binding buffer (20 mM Tris- HCl, 100 mM NaCl, 2 mM MgCl_2_, 5 mM KCl, pH 7.5 supplemented with 0.05% Tween-20). Aptamers were pretreated in the same way as described in BLI experiments and detected at 25 °C. At least three independent experiments were performed, and the data were analyzed to calculate the *K*_d_ value by MO.Affinity Analysis Software.

### 4.6. Preparation of Aptasensor

The SSA Sensor is considered to be a kind of super-sensitive and efficient sensor with great potential in the analysis of the molecular interactions [29]. The finally optimized STX aptamer 45e-1 was labeled with biotin and coupled to the chip surface coated with SSA, and prepared into an aptasensor. The stability and repeatability of the aptasensors were evaluated by the interaction between the sensors and STX in different concentrations and solvents. The aptasensors were used to detect the STX, which was prepared according to the concentration gradient (50–1600 ng/mL) in order to obtain the relationship between the STX concentration and the response value. Additionally, different concentrations of STX (100 ng/mL, 200 ng/mL, 500 ng/mL) in seawater (PH 7.5) from the Jinshan marine area of Shanghai were detected by means of aptasensors.

## Figures and Tables

**Table 1:** Average binding energy and its components obtained from the MM-GBSA calculation for the 45e-STX.

Contribution	∆E_VDW_	∆E_ele_	∆G_polar_	∆G_nonpolar_	∆G_total_
Energy (kcal/mol)	−25.02 ± 0.41	−381.64 ± 1.81	386.11 ± 1.59	−2.83 ± 0.03	−23.38 ± 0.53

**Table 2:** Detection of STX in seawater samples by aptasensors of 45e-1.

No	Concentration of STX (ng/mL)	Recovery (%)	CV (%)
1	100	106	4.3
2	200	110	6.7
3	500	97	2.4

**Table 3:** Comparison of different methods for the detection of STX.

Method		Sample	Selecting Method of Aptamer	Kd (nM)	Linear Range	Limit of Detection	Recovery (%)	References
**HILIC-MS/MS**		bivalve aquatic products, Standard addition	—	—	—	18–25 nmol/kg tissue	92–96	Thomas et al. 2017 [7]
		*Scomberomorus niphonius*, oyster, blood clam	—	—	—	2.9–4.3μg/kg	76.8–93.6	Zhuo, et al. 2013 [8]
**Precolumn oxidation HPLC**		rats’ brain homogenate	—	—	0.05–20 ng/mL	11.19 ± 0.11pg/20μL injection	59.5 ± 1.5 for total brain	Cianca et al. 2007 [9]
**Postcolumn oxidation HPLC-FLD**		mussel, clam, scallop, oyster	—	—	0.007–3.812mg STX.diHCl/kg	0.0038–0.0044mg STX.diHCl/kg	78.8–83.4	Veronica et al. 2016 [11]
					—	—	101–123	Jeffrey et al. 2011 [12]
**Direct ELISA**		human whole blood	—	—	0.010–1.0 ng/mL	0.02 ng/mL	—	Wharton et al. 2017 [6]
		dried human blood				0.06 ng/mL		
**Aptasensor**	1.Electrochemical aptasensor	mussels	Fixing saxitoxin	APT^STX1^ (3840)	0.9–30 nM	0.38 nM	63–120.5	Hou et al. 2016 [28]
		seawater	Fixing saxitoxin	APT^STX1^ (3840)	1–400 nM	0.92 nM	94.4–111	Qi et al. 2020 [56]
	2.Spectroscopic ellipsometry (SE) aptasensor	fish and shrimp	Fixing saxitoxin	APT^STX1^ (3840)	0.1–1000 ng/mL	0.11 ng/mL	—	Caglayan et al. 2020 [31]
	3.Competitive biosensor	shellfish, ribbon fish and water	Site-directed mutation and truncation of APT^STX1^	M30f (133)	100–800 ng/mL	0.50 ng/mL	101.4–107.3	Gao et al. 2017 [29]
	4.Fluorescenceswitch sensor	Shellfish			0–24 ng/mL	1.80 ng/mL	105.7–111.2	Cheng et al. 2018 [57]
	5.Colorimetric aptasensor	shellfish	Site-directed mutation and truncation of APT^STX1^	M30f (133)	78.13–2500 pM	42.46 pM	106.2–113.5	Zhao et al. 2021 [54]
		seawater and scallop	M30f Engineered by terminal hybridization	TF-M-30f (0.917)	0.1457− 37.30 nM	142.3 pM	98.21–114.1	Li et al. 2021 [55]
	6.LSPR aptasensor	mussels	GO-SELEX	50.75 ± 14.97	5–10,000 μg/L	2.46 μg/L	96.13–116.05	Ha et al. 2019 [35]
	7.Fluorescence assay	clam	MRGO-SELEX	STX-41(61.44 ± 23.18)	—	0.39 ng/mL	84.59–96.13	Gu et al. 2018 [34]
	8.Biosensor	seawater	IMC-SELEX	75a (136)	—	—	—	This work
		seawater		45e-1(19)	50–800 ng/ml	0.50 ng/mL	97–106	
	9.Electrochemical Ti3C2Tx	mussel tissues	SELEX	—	1.0–200 nM	0.03 nM	103	Ullah et al. 2021 [53]

## Data Availability

The data presented in this study are available in this article and supplementary materials.

## Supplementary Materials

The following supporting information can be downloaded at: https://www.mdpi.com/article/10.3390/toxins14030228/s1, Figure S1. (A) STX concentration and relative absorbance standard curve. (B) The mixed solution of STX and ssDNA was incubated with the GO and Ni-NTA column, the recovery rate of STX (3 ng/mL) in the effluent through GO and Ni-NTA column is 79.8% and 93.5%, respectively. (C) Adsorption capacity of Ni-NTA column (2 mL kit) to ssDNA. (D) Recovery ratio of ssDNA during IMC-SELEX; Figure S2. Secondary structure prediction of optimized sequences of STX-G4-45; Figure S3. Secondary structure prediction of aptamer 75, 75a, 75b, and 75c; Figure S4. Characterization of affinity and specificity of aptamer 45e for STX. The purple line represents the interaction curve of aptamer 45e with STX. The blue line represents the interaction curve of a random sequence with STX. The pink, brown, yellow lines represent the interaction curves of aptamer 45e with TTX, GTX1/4, neoSTX. All toxins are diluted to 5 µM with seawater, respectively; Figure S5. The final stable complex of 45e and STX; Figure S6. (A) The principle of the aptasensor for detection of STX. (B) Schematic of the working progress of the aptasensor; Table S1. Sequences of the random library and the G-quadruplex library.
