# Supplementary material for: A Novel SELEX Based on Immobilizing Libraries Enables Screening of Saxitoxin Aptamers for BLI Aptasensor Applications
Source: Toxins (Basel). 2022 Mar 21;14(3):228. doi: 10.3390/toxins14030228 (PMC8955768; doi:10.3390/toxins14030228)
Supplement: Supplementary file 1 [file toxins-14-00228-s001.zip › toxins-1611804-supplementary.pdf]

Supplementary Materials: A Novel SELEX Based on Immobilizing Libraries Enables Screening of Saxitoxin Aptamers for BLI Aptasensor Applications

Rong Zhou, Yun Gao, Chengfang Yang, Xiaojuan Zhang, Bo Hu, Luming Zhao, Han Guo, Mingjuan Sun, Lianghua Wang and Binghua Jiao

Table S1. Sequences of the random library and the G-quadruplex library

N-PrimerA1	GCCACACCCTGCCCTC	K_d (M)
N-PrimerA2	5′ Biotin-GCCACACCCTGCCCTC	/
N-PrimerB1	GAGGACACAGACAGACAC	/
N-PrimerB2	AAAAAAAAAAAAAAAAAAAA-Spacer18-GAGGACACAGACAGACAC	/
Random Library	GCCACACCCTGCCCTCNNNNNNNNNNNNNNNNNNNNNNNNNGTGTCTGTCTGTGTCCTC	/
1th Library	5′ Biotin-GCCACACCCTGCCCTCNNNNNNNNNNNNNNNNNNNNNNNNNGTGTCTGTCTGTGTCCTC	1.46×10^{3}
2th Library	5′ Biotin-GCCACACCCTGCCCTCNNNNNNNNNNNNNNNNNNNNNNNNNGTGTCTGTCTGTGTCCTC	2.11×10^{4}
3th Library	5′ Biotin-GCCACACCCTGCCCTCNNNNNNNNNNNNNNNNNNNNNNNNNGTGTCTGTCTGTGTCCTC	2.83×10^{5}
4th Library	5′ Biotin-GCCACACCCTGCCCTCNNNNNNNNNNNNNNNNNNNNNNNNNGTGTCTGTCTGTGTCCTC	1.62×10^{6}
5th Library	5′ Biotin-GCCACACCCTGCCCTCNNNNNNNNNNNNNNNNNNNNNNNNNGTGTCTGTCTGTGTCCTC	1.38×10^{6}
6th Library	5′ Biotin-GCCACACCCTGCCCTCNNNNNNNNNNNNNNNNNNNNNNNNNGTGTCTGTCTGTGTCCTC	9.08×10^{7}
STX-R -10	5′ Biotin- GCCACACCCTGCCCTCATCAAACATGCAAATCAAATAATAGTGTCTGTCTGTGTCCTC	1.18×10^{6}
STX-R-11	5′ Biotin- GCCACACCCTGCCCTCCACCCAGCAGTTTTTCATCCTCTAGTGTCTGTCTGTGTCCTC	3.05×10^{5}
STX-R-37	5′ Biotin- GCCACACCCTGCCCTCAACTCGGAAACGAATGTAAATATTGTGTCTGTCTGTGTCCTC	2.30×10^{7}
STX-R-40	5′ Biotin- GCCACACCCTGCCCTCATACAAAAAAATATTACGCAACTAGTGTCTGTCTGTGTCCTC	3.02×10^{7}
STX-R-42	5′ Biotin- GCCACACCCTGCCCTCCAACACCTGATATAGGATTCGGACGTGTCTGTCTGTGTCCTC	1.05×10^{6}
STX-R-57	5′ Biotin- GCCACACCCTGCCCTCAACCAGAAGTAAGAACTATCGCAGGTGTCTGTCTGTGTCCTC	5.80×10^{7}
STX-R-59	5′ Biotin- GCCACACCCTGCCCTCATATTGCAATAACCAAACCACCCAGTGTCTGTCTGTGTCCTC	7.23×10^{7}
STX-R-64	5′ Biotin- GCCACACCCTGCCCTCAAAGATAGTCAATTAACATACACGGTGTCTGTCTGTGTCCTC	3.22×10^{7}
STX-R-75	5′ Biotin- GCCACACCCTGCCCTCTAGGTGCGTTTCATATGAACTTTCGTGTCTGTCTGTGTCCTC	2.09×10^{7}
STX-R-78	5′ Biotin- GCCACACCCTGCCCTCTCACCAAAAGGAAAATATCAAAACAGTGTCTGTCTGTGTCTC	2.33×10^{7}
STX-R-80	5′ Biotin- GCCACACCCTGCCCTCCACACGTAACGTAAAATGGTCACAGTGTCTGTCTGTGTCCTC	2.16×10^{7}
75a	5′ Biotin-TAGGTGCGTTTCATATGAACTTTCGTG	1.36×10^{7}
75b	5′ Biotin-GCCACACCCTGCCCTCTAGGTGCGTTTCATATGAACTTTCGTGT	1.90×10^{7}
75c	5′ Biotin-TAGGTGCGTTTCATATGAACTTTC	NB
G-quadruplex Library	GCCACACCCTGCCCTCNNNGGNNNNGNNNNNNGNNNNGGGNNNGTGTCTGTCTGTGTCCTC	/
1th Library	5′ Biotin-GCCACACCCTGCCCTCNNNGGNNNNGNNNNNNGNNNNGGGNNNGTGTCTGTCTGTGTCCTC	1.01×10^{3}
2th Library	5′ Biotin-GCCACACCCTGCCCTCNNNGGNNNNGNNNNNNGNNNNGGGNNNGTGTCTGTCTGTGTCCTC	2.18×10^{5}
3th Library	5′ Biotin-GCCACACCCTGCCCTCNNNGGNNNNGNNNNNNGNNNNGGGNNNGTGTCTGTCTGTGTCCTC	1.47×10^{5}
4th Library	5′ Biotin-GCCACACCCTGCCCTCNNNGGNNNNGNNNNNNGNNNNGGGNNNGTGTCTGTCTGTGTCCTC	1.08×10^{6}
5th Library	5′ Biotin-GCCACACCCTGCCCTCNNNGGNNNNGNNNNNNGNNNNGGGNNNGTGTCTGTCTGTGTCCTC	9.87×10^{7}
6th Library	5′ Biotin-GCCACACCCTGCCCTCNNNGGNNNNGNNNNNNGNNNNGGGNNNGTGTCTGTCTGTGTCCTC	8.48×10^{7}
STX-G4-12	5′ Biotin-GCCACACCCTGCCCTCCACGGTTGTGATCTATGCGCAGGGTTGGTGTCTGTCTGTGTCCTC	5.22×10^{7}
STX-G4-22	5′ Biotin-GCCACACCCTGCCCTCCACGGAGGTGTAGGTTGTTACGGGTAAGTGTCTGTCTGTGTCCTC	9.89×10^{8}
STX-G4-27	5′ Biotin-GCCACACCCTGCCCTCCCCGGAAGTGTGTTAAGCTCTGGGACGGTGTCTGTCTGTGTCCTC	5.60×10^{8}

STX-G4-45	5′ Biotin-GCCACACCCTGCCCTCGGGGGCGCGGTTGATCGGAGAGGGCTAGTGTCTGTCTGTGTCCTC	4.26×10^{8}
STX-G4-47	5′ Biotin-GCCACACCCTGCCCTCGCTGGTAGAGACCTCAGCCTTGGGACCGTGTCTGTCTGTGTCCTC	7.58×10^{8}
STX-G4-51	5′ Biotin-GCCACACCCTGCCCTCACAGGCTCCGCTAGACGGACTGGGTTGGTGTCTGTCTGTGTCCTC	9.72×10^{8}
STX-G4-58	5′ Biotin-GCCACACCCTGCCCTCCCCGGTACCGAGACTCGCTTAGGGCGTGTGTCTGTCTGTGTCCTC	1.01×10^{6}
STX-G4-60	5′ Biotin-GCCACACCCTGCCCTCCTCGGTAATGTCCCATGTTACGGGTTAGTGTCTGTCTGTGTCCTC	9.50×10^{8}
STX-G4-62	5′ Biotin-GCCACACCCTGCCCTCTGCGGGCTTGAGTACGGAATAGGGCCTGTGTCTGTCTGTGTCCTC	6.39×10^{8}
STX-G4-67	5′ Biotin-GCCACACCCTGCCCTCGATGGCACCGGTGAGGGTGTTGGGAATGTGTCTGTCTGTGTCCTC	1.15×10^{7}
45a	5′ Biotin-ACACCCTGCCCTCGGGGGCGCGGTTGATCGGAGAGGGCTAGTGT	4.16×10^{8}
45b	5′ Biotin-CTCGGGGGCGCGGTTGATCGGAGAGGGCTAGTGTCTGTCTGTGT	3.83×10^{8}
45c	5′ Biotin-GGGGGCGCGGTTGATCGGAGAGGGCTA	NB
45d	5′ Biotin-CGCGGTTGATCGGAGAGGGCTA	NB
45e	5′ Biotin-CTCGGGGGCGCGGTTGATCGGAGAGGGCTA	2.12×10^{8}
45f	5′ Biotin-CTCGGGCCCGCGGTTGATCGGAGAGGGCTA	NB
45g	5′ Biotin-CTCGGGGGCGCCCTTGATCGGAGAGGGCTA	NB
45h	5′ Biotin-CTCGGGGGCGCGGTTGATCCCAGAGGGCTA	NB
45i	5′ Biotin-CTCGGGGGCGCGGTTGATCGGAGACCGCTA	NB
45e-1	5′ Biotin-CTCGGGGGCGCGGTTGATCGGAGAGGG	1.90×10^{8}
45e-2	5′ Biotin-CTCGGGGGCGCGGTTGATCGGAGAGG	3.06×10^{8}
45e-3	5′ Biotin-CGGGGGCGCGGTTGATCGGAGAGG	2.68×10^{7}

NB: No Binding

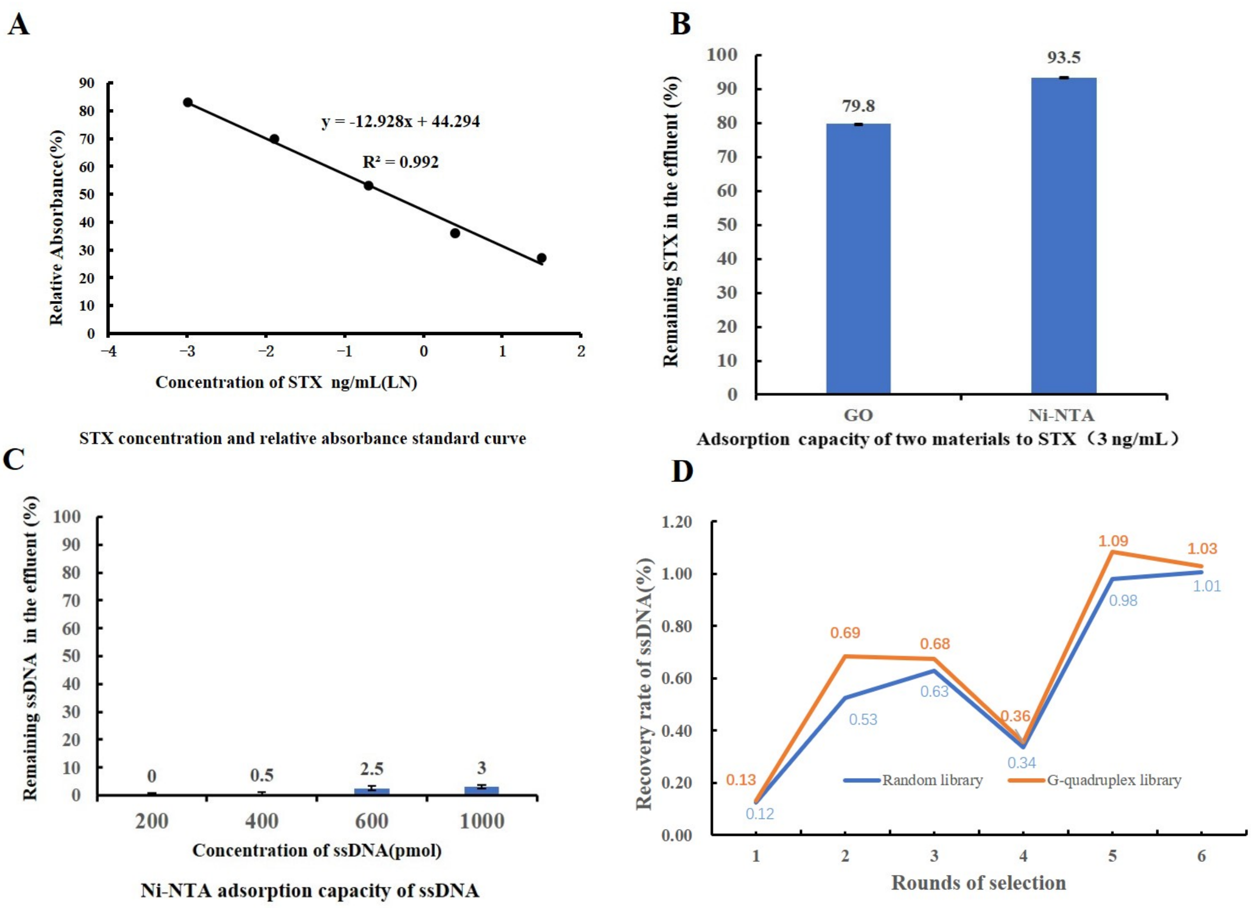

Figure S1. (**A**) STX concentration and relative absorbance standard curve. (**B**) The mixed solution of STX and ssDNA was incubated with the GO and Ni-NTA column, the recovery rate of STX（3 ng/mL）in the effluent through GO and Ni-NTA column is 79.8% and 93.5%, respectively. (**C**) Adsorption capacity of Ni-NTA column (2 ml kit) to ssDNA. (D) Recovery ratio of ssDNA during IMC-SELEX.

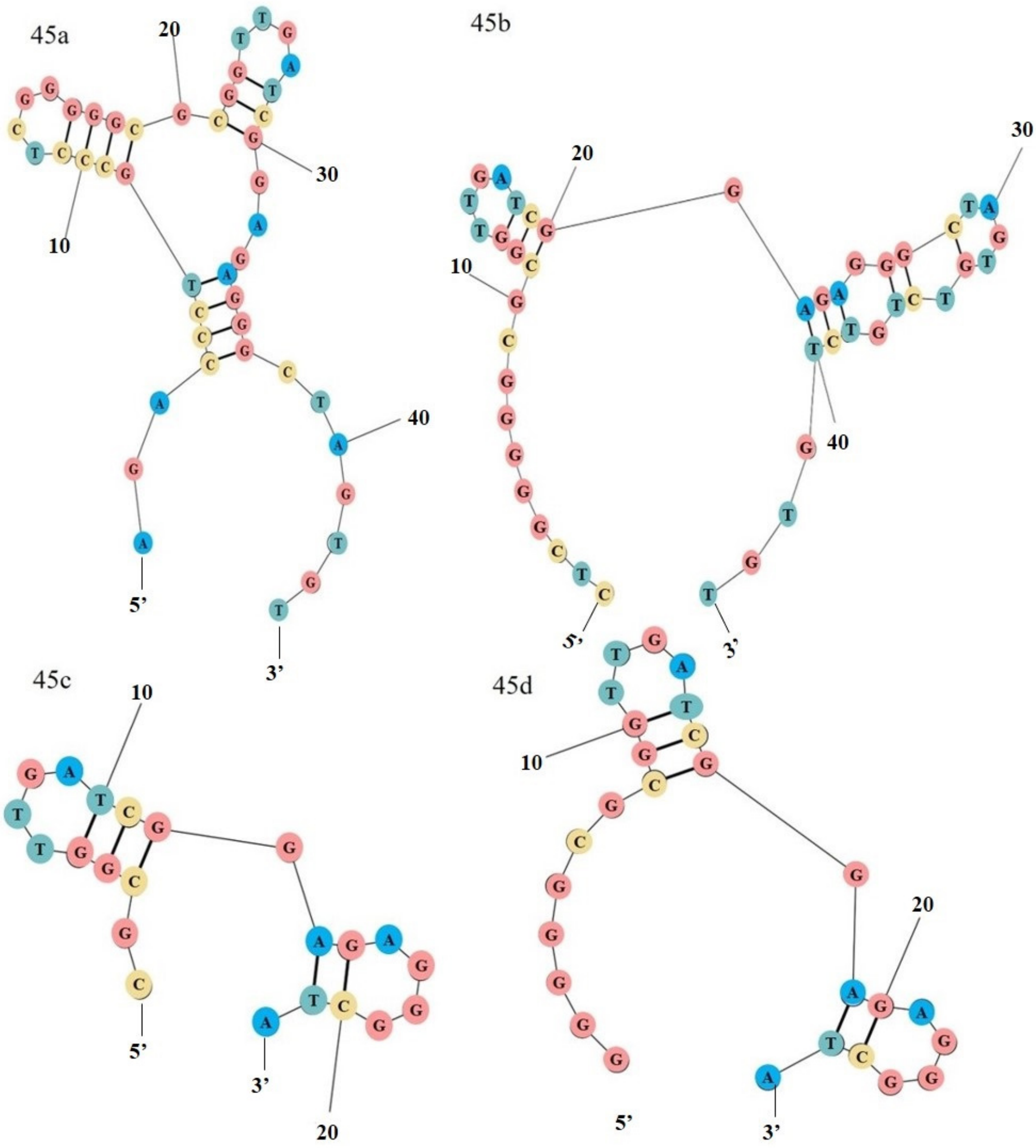

Figure S2. Secondary structure prediction of optimized sequences of STX-G4-45.

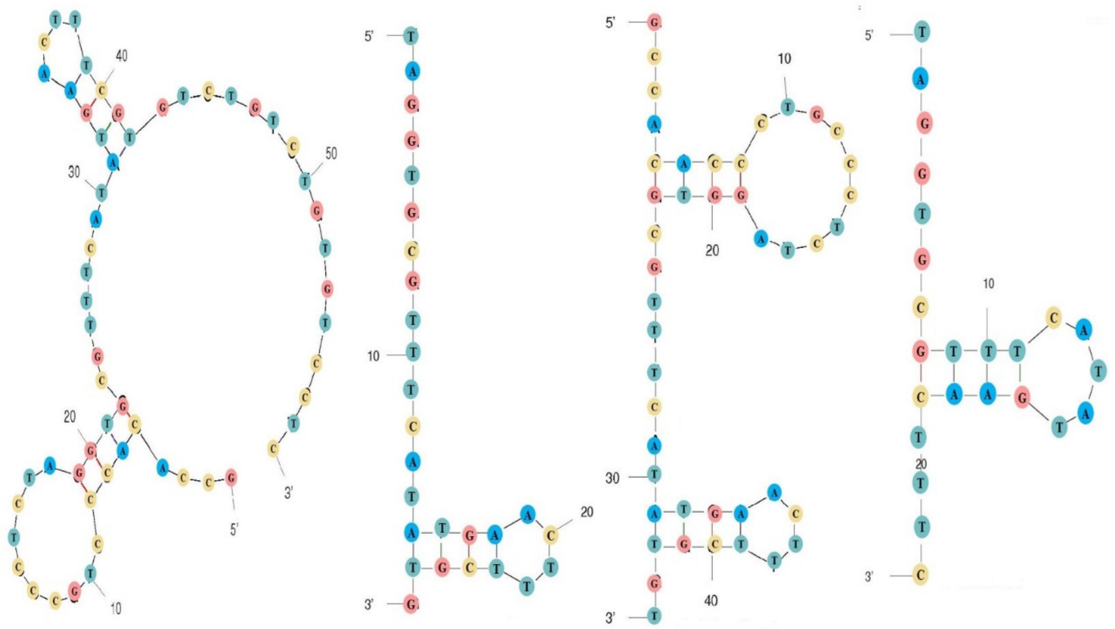

75 75a 75b 75c

Figure S3. Secondary structure prediction of aptamer 75, 75a, 75b, and 75c.

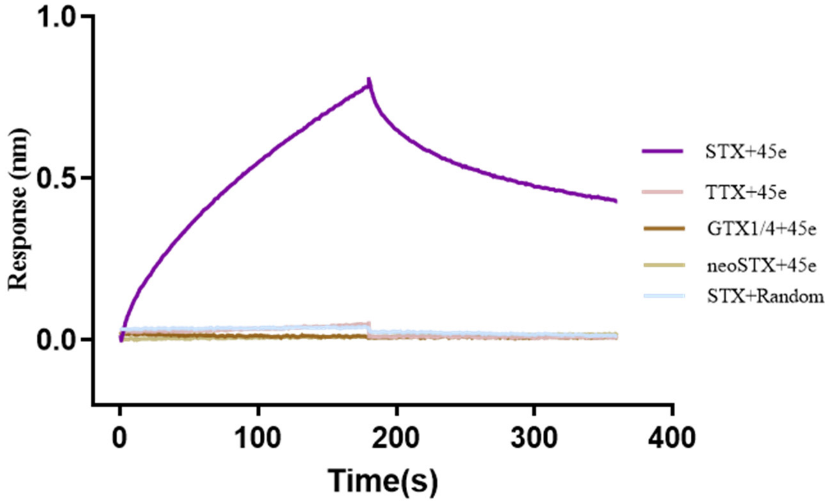

Figure S4. Characterization of affinity and specificity of aptamer 45e for STX. The purple line represents the interaction curve of aptamer 45e with STX. The blue line represents the interaction curve of a random sequence with STX. The pink, brown, yellow lines represent the interaction curves of aptamer 45e with TTX, GTX1/4, neoSTX. All toxins are diluted to 5 μM with seawater, respectively.

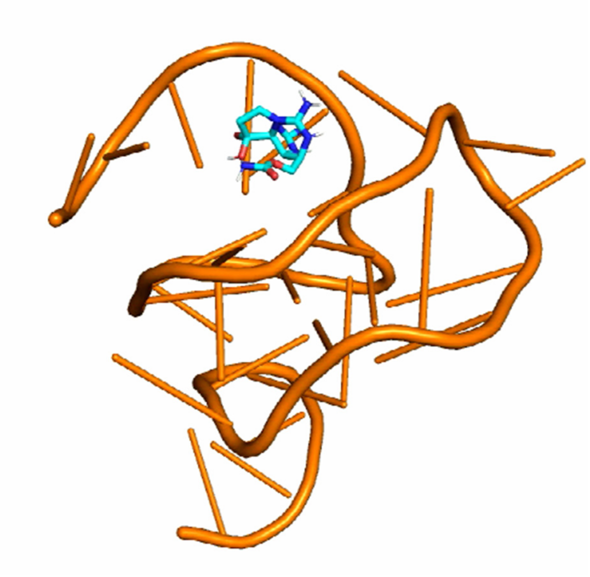

Figure S5. The final stable complex of 45e and STX.

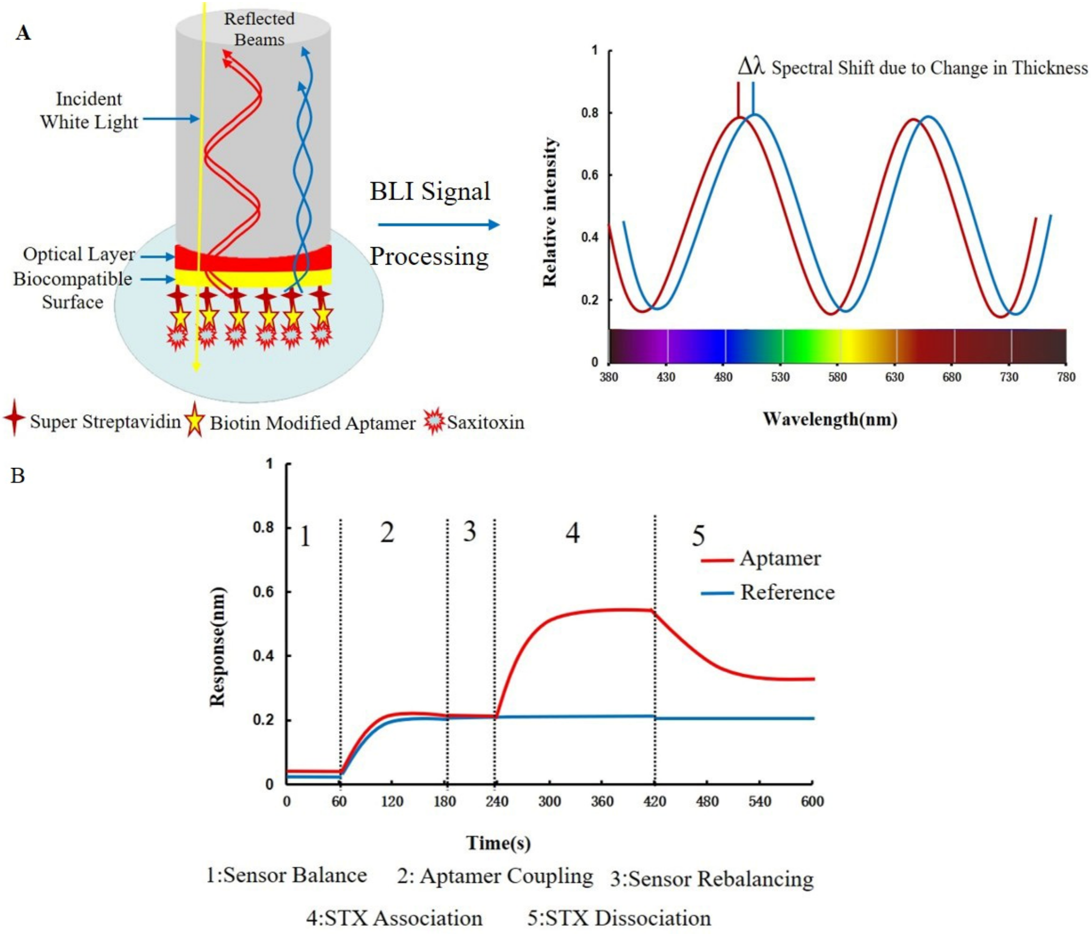

Figure S6. (**A**) The principle of the aptasensor for detection of STX. (**B**)Schematic of the working progress of the aptasensor.
